# The Dark Side of Moral Conviction—Integrating Political Psychology, Cognitive Science, and Neuroscience

**DOI:** 10.1111/nyas.70109

**Published:** 2025-10-13

**Authors:** Jean Decety, Michael S. Cohen, Qiongwen Cao

**Affiliations:** ^1^ Department of Psychology University of Chicago Chicago Illinois USA; ^2^ Department of Psychiatry and Behavioral Neuroscience University of Chicago Chicago Illinois USA

**Keywords:** conviction, dogmatism, metacognition, morality, political psychology, social neuroscience, valuation system

## Abstract

Morality is a pervasive characteristic of human societies, with social norms and codes of conduct defining acceptable and unacceptable behaviors across cultures. Our evolved moral sense facilitates group living by regulating interpersonal interactions and promoting cooperation beyond the bounds of kinship ties. Moral beliefs that are held with high certainty and perceived as absolute and universally applicable can motivate a strong commitment to justice and benevolent collective action. They also have a darker side. Moral conviction can foster dogmatism, intolerance, and punitive actions, including vigilantism and violence. This article integrates theories and empirical evidence from evolutionary social psychology, cognitive science, political psychology, and neuroscience to examine both the ultimate and proximate mechanisms of moral conviction. This interdisciplinary approach clarifies the functional architecture and potential deleterious consequences of moral conviction.

## 1 Introduction

Morality is a fundamental aspect of our humanity, contributing to large‐scale cooperation. The extent to which a person considers a given topic (e.g., gender equality, drug addiction, climate change, abortion, medically assisted suicide, or commercial meat production) to fall within the moral domain has wide‐ranging consequences. This is particularly the case when moral beliefs are held with strong conviction and perceived as objectively true and universally applicable. Such moral convictions can motivate people to engage in collective action and inspire courage to stand up against injustice, even at a personal cost. However, moral convictions also have a darker side. For instance, articles on highly moralized issues like racial equality or immigration are more likely to be shared on social media, even when the information is false, by both liberals and conservatives in the United States [1]. Moral convictions can fuel hate‐based violence and undermine civil discourse. Online platforms in particular are rife with hate speech targeting ethnicity, gender, and other social identities, contributing to the spread of extremist ideologies and causing tangible harm to their targets [2]. Importantly, once an issue is highly moralized, it is more likely to be perceived as an absolute belief and held with high confidence, making people unwilling to seek out corrective information and leading them to be dogmatic regardless of accuracy.

Most of the existing work on this topic has been conducted within social psychology, with a primary emphasis on the functions of moral conviction. In this article, we place greater focus on both the ultimate causes and proximate mechanisms underlying moral convictions, while also highlighting their harmful consequences. The dark side of moral conviction presents a paradox that is both intellectually intriguing and socially relevant. Drawing on theories and empirical findings from evolutionary anthropology, social psychology, cognitive science, political psychology, and neuroscience, we adopt an integrative approach across multiple levels of analysis to provide a comprehensive understanding of this phenomenon. This interdisciplinary perspective is particularly valuable, as some of the most interesting questions and insights emerge at the intersection of these different disciplines.

**BOX 1: Definitions of the concepts used**

- **Beliefs** are propositional attitudes with representational content and assumed veracity. Beliefs do not need to be conscious or linguistically articulated. They can be based on evidence, personal experiences, and cultural influences.
- **Brain valuation system** is a set of interconnected neural regions encoding subjective values. This circuit comprises the ventromedial prefrontal cortex (vmPFC), the orbitofrontal cortex, and subcortical regions, particularly the ventral tegmental area and ventral striatum, as key components. This system can assign values to different categories of objects, such as food, money, or abstract ideas, by a common neural currency model. This subjective value guides decision‐making and behavior.
- **Coalition**: Social groups, from small cliques of friends to entire nations, and from tribes to trade unions, exist because individuals are motivated to join and remain. Coalitions consist of individuals behaving in ways that enhance each other's welfare.
- **Ideology** is a set of organized beliefs about the proper order of society and how it can be achieved. Political ideology serves as a social force that organizes beliefs and attitudes regarding how society ought to be structured and how individuals ought to behave.
- **Information avoidance** is a refusal to access available information, even if it might improve decision‐making, because it is unpleasant or challenges one's existing views.
- **Metacognition** refers to knowledge about one's own thoughts and cognitive processes as well as the cognitive regulation involved in directing one's learning and decision‐making based on this knowledge.
- **Moralization** involves assigning moral significance to previously neutral preferences or actions.
- **Moral beliefs** are a specific subset of beliefs that involve judgments about what is right or wrong. These beliefs often reflect principles and values that guide behavior and shape social norms.
- **Moral conviction** refers to beliefs or attitudes that a person would describe as being related to core moral beliefs and to their fundamental sense of right and wrong. Beliefs and attitudes with a high level of moral conviction are perceived as absolute, objective, and universal.
- **Motivated reasoning** occurs when individuals use their reasoning abilities to selectively interpret, weigh, and dismiss evidence in a manner that confirms their beliefs.
- **Social norms** can be categorized as either injunctive or descriptive. The former are behavioral expectations that are backed by social or material sanctions, while the latter are simply regularities of behavior, what most people do in a given situation.
- **Values** are motivational forces (attraction toward or repulsion away), representing potential energy and desires that guide decisions and motivate actions.

We begin with a brief overview of the ultimate explanations of morality, emphasizing its strategic role in group dynamics. Next, we describe the moralization process, through which neutral preferences or attitudes are transformed into values, acquiring emotional intensity, and motivating behavior. We then examine the defining characteristics of moral conviction (i.e., strongly held moral beliefs) and their negative functional consequences, such as an unwillingness to compromise. We further explore the domain of metacognition to account for cognitive rigidity often associated with moral convictions. In the final section, we discuss the cognitive and neural mechanisms underlying information avoidance, which may underlie resistance to challenges against morally held beliefs. We conclude by highlighting how this integrative perspective offers a deeper and comprehensive theoretical framework for understanding the mechanisms underlying moral conviction and metacognition (see Box 1 for definitions).

## 2 The Evolution of Morality and Coalitional Dynamics

Sociability is a biological adaptation to socioecological pressures [3]. Group living offers better protection against predators, facilitates access to resources such as information, food, and mates, as well as raising offspring, all of which are crucial for reproduction and survival [4]. Species that live in groups tend to have longer lifespans than solitary ones [5]. Ecological pressures made humans particularly interdependent for survival, instilling a strong need for social connection. These pressures also led them to seek autonomy, enabling our ancestors to distinguish themselves within groups, thereby improving their chances of reproduction and social status. Our predisposition for social preferences and cooperation has emerged through natural selection, primarily due to the benefits it provided to our ancestors living in large groups [6]. This required complex systems of social evaluation to distinguish individuals who could be trusted, that is, those who were likely to cooperate, from those who would not [7]. Humans have a unique ability to create cultural structures that establish social norms—explicit and implicit—to regulate acceptable behaviors and goal achievement, enforcing them through institutions designed to assess the acceptability of individuals’ behaviors and assign consequences for violating these norms [8].

The standard evolutionary view of morality holds that moral behaviors and intuitions have evolved through natural selection. Our moral sense rests on a collection of capacities that developed in *Homo sapiens* as a means for fostering cooperation within groups of genetically nonrelated individuals to overcome challenges from both inside the group and outside the group [9, 10, 11, 12, 13]. Morality serves as a powerful incentive to engage in cooperation among one's group members that is not rooted in pure self‐interest.

Many moral evaluations focus on actions that involve some form of harm, such as loss of life, physical harm, loss of rightful property, invasion of privacy, or other threats to autonomy [14]. At the core of moral judgment, some authors have proposed a broad cognitive model of harm that can generally be defined as the perception of an intentional agent causing damage to a vulnerable recipient. This harm‐based cognitive framework is intuitive, rooted in innate evolved mental processes, yet also shaped by cultural learning, allowing for various moral perspectives across societies [15]. In support of this theory, third‐party harm aversion emerges early in ontogeny and forms a necessary foundation for morality [16]. Perceiving another person being harmed elicits a rapid neurophysiological response as early as 62 ms post‐stimulus in the right posterior superior temporal sulcus (also known as the temporoparietal junction, rTPJ), a region that plays a critical role in social perception by detecting and predicting the intentions underlying actions [17]. Studies using high‐density electroencephalography and intracranial electrodes demonstrated that perceived harm induces activity in the amygdala, a region that detects salience and valence, within 200 ms, and later activates the vmPFC, which encodes the value of stimuli [17, 18]. Perceiving another person being intentionally harmed leads to increased pupillary dilation, a reliable index of autonomic arousal, which correlates with neural response in the amygdala and rTPJ [19]. Importantly, this response is observed very early in ontogeny. For instance, a study showed preverbal infants aged 12−24 months video clips of an agent performing either interpersonal harm or help while measuring event‐related potentials, power density, and pupillometry [20]. Infants differentiate between interpersonal harm and interpersonal help within 300 ms after stimulus onset. This fast reaction reflects an automatic allocation of attentional resources and a negativity bias.

Another theoretical view, less centered on harm, posits that morality relies on a collection of biological and cultural solutions that evolved to address the problems of cooperation recurrent in human social life [21]. Morality regulates interpersonal exchanges, facilitates coexistence and cooperation, channels aggression, and helps achieve a balance when individual interests conflict with collective interests. Natural selection favored adaptations that enable the realization of opportunities for mutually beneficial non‐zero‐sum interactions that social life affords. In short, we are motivated by morality because it is advantageous at the individual level [11, 12].

The evolution of morality may have relied on group‐level natural selection that drew on intragroup cooperation and intergroup hostility [13, 22]. Humans have always relied on the support of both kin and unrelated individuals to survive and thrive. Such support has been indispensable across various domains of social and economic life. Throughout human evolution, reproductive success has depended on the propensity of genetically unrelated individuals to support one another, form groups, and adopt shared social identities [23]. Selective socioecological pressures have fostered an adaptation for coalitions, in the form of (heuristic) programs that promote forming, maintaining, joining, supporting, recognizing, defending, exploiting, resisting, subordinating, distrusting, hating, opposing, and even attacking coalitions [24]. Coalitions are constituted as groups of individuals perceived by themselves and others as sharing a social identity, acting as a unit, defending common interests, and having shared mental states, or a sense of “*we*”*‐ness*. Our species has evolved in a context of intense intergroup competition, where groups composed of loyal members were more likely to succeed than those with less cohesive members [25]. Consequently, selective pressures have shaped our psychology, giving rise to a disposition toward tribalism. Group loyalty and its associated cognitive biases are present in all human societies, often distorting beliefs in favor of one's coalition. The primary function that drove the evolution of coalitions is the amplification of the power of its members in conflicts with nonmembers [26]. In addition, securing group unity requires punishing those who fail to conform to prevailing norms.

The coalitional psychology framework suggests that intentionally harming others can be perceived as more or less commendable depending on group dynamics and the relational and cultural context [27]. Prejudices can range from verbal aggression to large‐scale group conflict. Actions are considered good, just, equitable, virtuous, or morally correct when they align with specific sociorelational contexts, and wrong or unjust when they do not. For instance, people are more willing to accept aggressive, dishonest, or otherwise antisocial behaviors directed toward an out‐party politician than toward people encountered in everyday life, and this tendency is stronger in those who report having authentic and deep antipathy toward the political outgroup [28]. Even violent actions can be morally justified if they uphold social norms within a family, ethnic group, tribe, religion, or nation [29]. Actions that violate these relational models are considered immoral. For relationships to thrive, individuals must strike a balance between competing motivations that regulate behavior and maintain social order.

Therefore, the core of our moral psychology consists of grounds for evaluating and orienting our judgments and behaviors and those of others (including speech, emotions, attitudes, and intentions) with reference to prescriptive models. Behaviors that do not conform to relational prescriptions are considered moral transgressions and arouse emotions such as guilt, shame, disgust, envy, or indignation. These emotions motivate sanctions, including apologies, repairs, and rectifications, and the modulation or termination of the relationship.

Consistent with coalitional psychology theory and the social heuristic exchange theory, a substantial body of research demonstrates that individuals derogate deviant ingroup members to protect the group from threats to its social identity—a phenomenon known as the “black sheep effect.” People exhibit a stronger preference for exclusionary punishments against deviant ingroup members due to heightened perceptions of ingroup threat [30]. Third‐party experimental economics punishment games further demonstrate this effect, showing that noncooperative in‐group members are judged more harshly and punished more severely than out‐group members [31]. The black‐sheep effect is most pronounced when group‐based motivational concerns are activated, such as strong group identification, perceived threats to the group's reputation, or the belief that members share similar characteristics, especially in response to violations of group‐specific norms [32]. Because immoral behaviors by ingroup members jeopardize the group's reputation, it triggers utilitarian exclusion‐oriented motives for punishment.

Overall, behaviors motivated by morality rely on adaptations that evolved to facilitate group living and cooperation. Our moral psychology is thus, in part, a natural extension of the cognitive mechanisms underlying the formation of coalitions, as well as adaptations for social assortment and exclusion.

## 3 The Process of Moralization

Moralization can be conceived as the *degree* to which moral relevance is attached to given actions (e.g., social loafing), issues (e.g., abortion), entities (e.g., an octopus), nonsentient objects (e.g., an antique sculpture), and groups (e.g., transgender people) [33]. Factors that contribute to moralization can be emotional, such as feeling “moral emotions” about a topic (e.g., disgust, outrage, or guilt), or cognitive, when a newly moralized issue is linked to an existing moral belief, or both [34]. Associations between beliefs, attitudes, and subjective values act as a critical signal because values guide decisions and motivate action, as discussed further below. A moralized belief constitutes a powerful motivational force, directing behavior toward desirable outcomes, and serving as a compelling mandate regarding how to behave in specific circumstances [35]. Moralization elicits stronger emotional responses such as anger and disgust, mobilizes support for related causes, and influences the transmission of these beliefs between individuals [36, 37, 38, 39]. Moralization can occur at either the individual level or the cultural level [40]. Group norms, both descriptive and injunctive, play a role in moralization. Information regarding behaviors that constitute the descriptive norm profoundly influences the degree to which a behavior is perceived as moral. Importantly, this descriptive conception of moralization is different from a normative conception [41].

At the core of the moralization process lies the role of values. Values are motivational constructs that express what individuals consider important [42]. They shape our attitudes toward what is desirable or undesirable, attractive or repulsive, right or wrong. More broadly, value reflects the strength and direction of motivational forces that guide behavior toward preferred outcomes. It represents the intensity of attraction toward or aversion against an object, action, or idea [43]. This ability to assign value in some manner and to make decisions based on those valuations is a fundamental property of all biological organisms. This process motivates and guides their actions. Valuations put meaning into neural computations. It functions as a cost‐assignment mechanism that determines what the organism should “care” about, a signal that the organism must pay for as an up‐front energetic investment [44]. The amount of energy a computation should get is a measure of its value to the overall goal. From the perspective of his field theory, Lewin [45] proposed that values function as power fields. They represent potential energy or latent desire. When activated by opportunity or threat, they produce goals, conceptualized as force fields or kinetic energy or actual desire [46]. In this framework, goal‐directed motives operate as vectors within a force field, reflecting the individual's drive to approach positively valenced goals or avoid negatively valenced outcomes.

Both nonmoral and moral values are substantially influenced by social ecology and culture that shape, for instance, how we view beauty or charitable donations [47]. Importantly, moral values are special in the sense that they enable individuals to reap the benefits of social existence [48, 49]. They tend to possess intrinsic properties (valuable in their own right) and, therefore, are immune to being exchanged for mundane secular resources, such as money [50]. While moral values are conceptually distinct from nonmoral ones, their neural representations largely overlap with those involved in other types of values. In line with the neural reuse theory [51, 52], research in cognitive neuroscience and neuroeconomics indicates that the subjective values of a wide variety of pleasurable and aversive outcomes are encoded by a domain‐general system that also represents nonmoral values. The neural circuit engaged in value‐based decisions (ventral striatum and vmPFC) thus not only plays a role in evaluating primary choices that fulfill basic biological needs such as food, water, and sex, but also secondary choices including culturally mediated reinforcers like money and praise [53]. Importantly, secondary reinforcers can, over time, acquire the characteristics of primary rewards; cultural artifacts can develop neural signatures associated with the fulfillment of biological needs [54, 55].

Humans have a special capacity to value arbitrary objects that can become high‐status rewards. For instance, selective activation of the reward circuit was detected in devoted Danish Christians who were prompted to pray silently while being scanned with functional magnetic resonance imaging (fMRI) [56]. This pattern of neural activity was only found in participants who pray regularly. In another study with Mormon participants, the sensation of “feeling the holy spirit,” which is central to their devotional practice, was reliably associated with activation of the ventral striatum, vmPFC, and prefrontal attentional circuitry [57]. Interestingly, the activation of the striatum preceded the peak of spiritual feeling by 1−3 s. The association of abstract ideas and the brain reward circuit may interact with prefrontal processing of attention and emotions, suggesting a mechanism by which religious or doctrinal concepts may become intrinsically rewarding and motivate people's behaviors.

More generally, moral judgment and decision‐making share several common components, such as value representations and reinforcement learning. It has been proposed that action‐based and outcome‐based values are two critical representations in a dual‐system framework used to account for diverse moral evaluations [58]. According to this framework, the motivations for moral evaluations can be valuing the intrinsic status of actions (e.g., “I don't lie because lying is wrong”) or valuing the expected consequences of actions (e.g., “If I lie, it will cause the person harm”). One neuroimaging study investigated functional differences based on Kohlberg's moral development theory [59], which distinguishes three levels of moral reasoning: a pre‐conventional level (motivated by self‐interest), a conventional level (motivated by adherence to social order, rules, and laws), and a post‐conventional level (motivated by social contracts and universal ethical principles). The study found significant differences in brain activity between individuals at the post‐conventional level of moral reasoning and those at the pre‐conventional or conventional levels [60]. Specifically, individuals who rely more on high‐level post‐conventional reasoning exhibited increased neural activity in the reward system, both at rest and during decision‐making tasks. While the precise nature of this association is not specified, this result underscores the importance of reward in moral reasoning.

Moral values play a significant part in shaping our identity and influencing our perceptions, decision‐making, and social behavior. Moralizing beliefs and attitudes increase their strength in certainty and importance, which, in turn, motivates social commitment. Presenting an action within a moral framework tends to result in more intense judgments and encourages prosocial behavior. In a series of experiments, participants were asked to evaluate a range of everyday actions (e.g., recycling) in either moral terms (e.g., morally good or bad) or nonmoral terms (e.g., pragmatically, or hedonically good or bad) [61]. The results revealed that moral evaluations led to faster responses, more extreme judgments, and greater universal prescriptiveness compared to nonmoral evaluations of the same actions. These findings support the hypothesis that moral framing promotes absolutist thinking, leading to more categorical, polarized, and binary judgments.

The literature reviewed here suggests a gradient model of moralization—that is, the degree to which moral relevance is attached to issues, actions, principles, or entities on a continuum rather than being a categorical distinction [62]. Morally convicted beliefs, as discussed below, tend to exhibit high levels of moralization, though the extent to which an issue is morally convicted also varies noncategorically. Specifically, while moralization describes the degree of transformation from nonmoral to moral, *moral amplification* reflects increases in the strength of moralization once moral value has already been attached. This concept maps onto changes in levels of moral conviction [33].

## 4 Characteristics of Moral Conviction

Moral beliefs and attitudes are distinct from other beliefs and opinions in several ways, especially when held with strong conviction. These characteristics—objectivity, emotion, and intrinsic value—are flexibly reinforced by each other. Moral convictions are grounded in core beliefs about fundamental right and wrong and are considered to be standards applicable to all human beings, eliciting monitoring and judgment of their actions [63, 64]. They resist being processed through a cost−benefit analysis [65], and predict important social and political consequences.

‐ **Objectivity**: Moral convictions are perceived as objective facts that should be universally held, independent of the beliefs and preferences of individuals and cultures [66]. Perceived consensus exerts a causal role on objectivity [67]. Furthermore, when perceived consensus about a given moral issue is high, judgments are made with higher confidence. This occurs both due to the direct effects of social influence and to internal sampling processes in decision‐making that are distinct from social pressure [68]. Endorsement of moral objectivism provides people with a rigid, unambiguous, and definitive set of rules and expectations that are applicable across diverse social situations and contextual circumstances, which seems particularly valuable for individuals who value order and structure in the chaotic social environment [69].
‐ **Emotions**: People experience moral beliefs as inherently motivational and emotionally charged, and this is especially the case for moral convictions, which are associated with higher levels of autonomic nervous system physiological excitability [70], as well as strong positive and negative emotions [39, 71]. These effects have real‐world implications; for instance, one study using a large sample of messages on Twitter (*n* = 563,312) found that the presence of moral‐emotional words in messages increased their transmission by approximately 20% per word [36].
‐ **Intrinsic value**: Strong moral beliefs afford respect, caring, and consideration for a particular issue or object, irrespective of its instrumental or utilitarian value [72]. In other words, valuing it for what it is, not only for what it does. Such values confer direct moral standing and motivate people to protect them for reasons other than their usefulness or as means to another end. Decisions and behaviors that uphold moral convictions are experienced as highly rewarding [14, 73, 74]. In this way, moral convictions have a higher potential for action than views reflecting nonmoral preferences or social conventions, regardless of their strength [75].

Overall, moral convictions can be viewed as a set of implicit and explicit representations that incorporate cognitive, emotional, and value‐based dimensions. Cognitively, they involve the belief that one's moral position reflects a universal and objective truth. Emotionally, they reflect increased intensity, with stronger emotions associated with greater motivational force, further amplified by the intrinsic reward of acting in accordance with one's moral values. Moral convictions also help define not only who individuals are, but also who they consider to be part of their moral ingroup or outgroup [76].

While moral convictions can yield both beneficial and detrimental outcomes, we primarily focus here on their negative consequences, precisely because these effects are often overlooked or may seem counterintuitive, making them particularly compelling to examine.

## 5 Adverse Consequences of Moral Conviction

Moral convictions can inspire activism and social progress, but they can also give rise to dogmatism, intolerance, opinion polarization, and political division [77, 78]. In the United States, disputed issues, including fossil fuel consumption, vaccination, illegal immigration, gender‐affirming care for minors, and public spending to reduce poverty, hold a prominent place in political discourse. Moral commitments shape perception, motivation, and reasoning, often reinforcing myside and confirmation biases. These effects are particularly pronounced in politics, where high stakes and coalitional dynamics drive moralized beliefs [28, 79]. Political contests determine the distribution of valuable resources, including wealth, power, and prestige. Victorious groups gain control of cultural narratives and governmental institutions, leveraging them to advance their coalition's interests, often at the expense of opposing groups [25].

Studies in political psychology indicate that, in the United States, Democrats and Republicans have grown to dislike and distrust each other in recent decades, a phenomenon known as affective polarization [77]. The adverse effects of affective polarization become evident as partisans engage in uncivil political discourse online, avoid social interactions with opponents, reject bipartisan cooperation, and experience dissonance at the prospect of even listening to opposing political views [80]. This is particularly the case in political contests, where individuals across the political spectrum often believe their positions on issues are correct, denigrate or stereotype those who disagree with them, engage in punitive actions, and express revulsion toward individuals perceived as violating their moral values [81]. Another major concern is that partisan animus spills over and affects behaviors and attitudes outside the political realm, such as romantic relationships, marriages, and social relationships more broadly [80]. Survey data from national samples, including a measure of propensity to moralize (a battery of questions that evaluate respondents’ level of moral conviction on different issues), shows that individuals who express a higher propensity to moralize political issues display a wider gap in partisan affect, more social distance, incivility, anger, and antagonism toward people from the opposing party [82]. These impacts of moral conviction render people unwilling to listen to opposing views or to compromise, thus hindering the coordination among various groups with competing interests that a functional democracy needs.

The fact that political disagreements have become more moralized in recent decades likely contributes to the high level of affective polarization and dysfunction in American politics [83, 84, 85]. Moralized political rhetoric exacerbates moral conflict and affective polarization, for instance, when economic and cultural anti‐immigrant claims are framed in moral terms [86]. Social media amplify these dynamics by amplifying posts that use moral‐emotional language [36], and those derogating outgroups [87]. Politicians who use moral appeals to show allegiance to their own political tribe at the expense of the other side are more likely to get attention and support from voters. This process reduces incentives for candidates to develop and enact policies that will improve the lives of their constituents in concrete ways.

Specific harmful effects of moralization on political discourse have been demonstrated in numerous recent studies. Several studies have shown that moralized language predicts hate speech on social media. For example, one study collected three datasets consisting of *N* = 691,234 social media posts and ∼approximately 35.5 million corresponding replies from Twitter, authored by societal leaders across politics, news media, and activism [88]. The authors employed textual analysis and machine learning to investigate whether moralized language present in source tweets is associated with differences in the prevalence of hate speech in the corresponding replies. The results indicate that across all datasets, higher frequencies of moral and moral‐emotional words predicted a higher likelihood of receiving hate speech. Moralized issues are also more likely to be associated with hate than even strongly disliked targets, both in laboratory studies and in the real world on social media [89]. Similarly, speeches and texts from Nazi Germany, as well as modern‐day hate speech on the social media site Gab, tend to target outgroups with moral language implying that the group is impure and polluting [2]. The same study found that hateful speech in more everyday contexts often involves moral language around disloyalty.

The use of moral rhetoric on social media has been associated with violence in the real world. Extreme movements can emerge through social networks, where the activity of others influences people's perceptions of those they identify with and who share their beliefs, leading to an impression of consensus. For example, an analysis based on 18 million tweets posted during the 2015 Baltimore protests against police brutality showed that the number of hourly arrests made during the protests was associated with the number of moralized tweets posted in previous hours [90]. Tweets containing moral rhetoric nearly doubled on days when clashes among protesters and police became violent. Less extreme than violence but still harmful is the spread of false information consistent with one's own point of view, which is linked to moralization across a range of issues such as gun control, vaccination, gender, and racial equality [1, 91]. Partisans on either side of the American political spectrum also show less empathic concern toward opposing‐party members even when the cause for empathy is entirely nonpolitical (e.g., a sprained ankle) [92]. This effect was mediated by views of out‐party members as immoral, and liberals show this bias more strongly than conservatives. Thus, moral motivations are associated with a range of hateful reactions and false narratives by which people attempt to hurt targeted outgroups.

When individuals hold beliefs based on strong moral values, they are less willing to consider alternative viewpoints or evidence that challenges their position and are more likely to engage in motivated reasoning [93]. They selectively seek out information that supports their beliefs while avoiding information that contradicts them, particularly when evaluating scientific findings perceived as morally offensive [94, 95]. This is also evident among scientists. A study reported that social psychologists who believed it would be harmful to disseminate research on genetic contribution to sex differences were also less likely to accept that such contributions might exist [96]. One compelling case is the debate over whether controversial scientific findings that could potentially be used to harm vulnerable groups should be censored. A series of preregistered studies with representative U.S. samples examined how individuals react to such findings. Participants were presented with brief discussions of scientific findings with potentially contentious implications, such as research suggesting that female protégés benefit more from male than from female mentors, or findings indicating no evidence of racial discrimination against ethnic minorities in police shootings [97]. Across studies, participants tended to overestimate public support for harmful reactions, such as banning the research, while underestimating constructive responses, such as investing in programs to support the affected groups. Moreover, individuals who found the scientific findings offensive exhibited greater difficulty in understanding them.

Contrary to popular narratives, for example, in conservative‐leaning media, scientific censorship from the political left has not predominantly been driven by authoritarian bureaucrats. An analysis of historical and contemporary evidence regarding the social, psychological, and institutional causes and consequences of scientific censorship reveals that scientific censorship is often motivated by moral concerns, particularly benevolence toward peer scholars, and prosocial concerns for the well‐being of human social groups, especially those viewed as vulnerable based on gender, race, or sexual identity [98]. While these findings do not dismiss the legitimate prospect that accurate scientific findings could be misused to cause harm—an ethical consideration that editors of scientific journals may need to address—they highlight the importance of critically evaluating moral intuitions. Decision‐makers must be cautious not to overstate the risks of such research, as censorship can, at times, be counterproductive by inadvertently amplifying attention or conferring legitimacy to the content in question. Those on the political right in the United States have also recently taken advantage of the backlash against such censorship to justify a broader campaign against all diversity, equity, and inclusion. This backlash is causing greater harm to marginalized people, demonstrating an additional type of risk.

Another apt example is how moral conviction about gender bias in academic hiring can sometimes obscure rational analysis. The underrepresentation of women in academic science is often attributed, both in scientific literature and in the media, to gender bias in hiring. While academia has been historically unfair to women, the situation has improved significantly in recent decades. Both available data on tenure track hiring and experiments show this improvement. For instance, National Research Council data from 1999 to 2003 indicate that at R1 research‐intensive universities, across fields such as civil engineering, physics, mathematics, chemistry, biology, and electrical engineering, women who applied for tenure‐track positions were more likely to be hired. Several studies over the past 20 years have shown a similar trend. For instance, Williams and Ceci conducted national randomized experiments and validation studies on 873 tenure‐track faculty (439 male, 434 female) from biology, engineering, economics, and psychology at 371 university colleges from 50 U.S. states and the District of Columbia [99]. Results revealed a 2:1 preference for women by faculty of both genders across both math‐intensive and non–math‐intensive fields, with the single exception of male economists, who showed no gender preference. An adversarial collaboration examined the vast, contradictory scholarly literature on gender bias in academic science from 2000 to 2020 [100]. The authors found no compelling evidence of widespread bias against women in four of six domains studied within academic tenure‐track science (hiring, journal acceptances, U.S. grant funding, and letters of recommendation) and some evidence of bias in two domains (salary and teaching ratings), albeit with qualifications. Despite these empirical findings, the dominant narrative in prestigious scientific outlets and popular media continues to emphasize hiring discrimination against women. A study suggests that moral commitment influences how individuals interpret findings about gender discrimination [101]. The authors ran seven preregistered online experiments in France with 3570 participants to investigate their evaluations of scientific reports of hiring discrimination in academia. Results show that the more people were morally committed to gender equality, as measured by a 3‐item scale reflecting perceptions of the issue as a moral imperative and identity‐defining, the more likely they were to judge such research, its methods, and results as convincing. Importantly, the association between moral commitment and evaluations was not reducible to prior beliefs. However, a moral commitment to gender equality was also associated with an increased likelihood of fallaciously inferring discrimination against women when the evidence contradicted this conclusion.

Overall, morality plays a crucial role in shaping group‐based identity and esteem, reciprocally influencing how individuals evaluate their in‐groups and respond to out‐groups. Moral conviction motivates people to engage in and pay more attention to just causes, such as protecting vulnerable groups. However, harm concerns may cause errors in perception and undermine cost−benefit analysis. Furthermore, when others contravene deeply held moral expectations, those who are morally convicted experience strongly adverse emotional reactions. Although people who take extreme actions for a cause (e.g., marching with tiki torches at the 2017 Unite the Right rally, bombing abortion clinics, or using shock tactics like throwing soup at the Mona Lisa to raise attention to climate change) may have very different ideological orientations and motivations, those willing to take extreme actions for a cause likely share common psychological mechanisms [102]. Motivated by deeply ingrained moral values, these individuals are willing to accept radical actions when they serve their purposes. It is important to note that radical actions are often perceived as less legitimate and effective than conventional actions [103].

## 6 Neural Mechanisms of Moral Conviction

There is no specific neural architecture underlying moral values compared to nonmoral values. Instead, both are encoded by a domain‐general system that evolved in vertebrates to track value representations regardless of the nature of the reward and across a variety of positive or negative outcomes. A network of interconnected regions, including the ventral striatum, vmPFC, and the lateral prefrontal cortex, are implicated in value integration [104]. Additionally, other regions, such as the amygdala, anterior insula, and anterior cingulate cortex (ACC), are often found to be activated by reward and punishment signals [53]. Moral decisions require the engagement of nodes outside the valuation system, specifically signals processed in the medial prefrontal cortex and temporoparietal junction, regions that represent and understand the actions, intentions, and emotions of other conspecifics [105, 106, 107]. Translating moral norms into moral behavior also involves changes in functional connectivity between the dlPFC and the valuation system [108, 109].

Several neuroimaging studies have begun to investigate the neural mechanisms underlying moral conviction. Non‐negotiable, inviolable, and nonutilitarian values, referred to as sacred values in anthropology [110] or protected values in economics [65], seem to be represented and processed by neurons in the orbitofrontal cortex, especially in vmPFC and vlPFC subdivisions. In one fMRI study, participants were scanned while presented with personal values that ranged from the mundane to the sacred [111]. Passively processing sacred values was associated with activation of the vlPFC compared to processing mundane values [112]. Neural activity in this region in response to sacred values also negatively correlated with individual differences in conformity, that is, the degree to which the proportion of the population shown to hold a given view influenced participants’ willingness to disavow their initial values. Thus, vlPFC activity is associated with unwillingness to adjust one's viewpoints in response to social influence.

**FIGURE 1:**
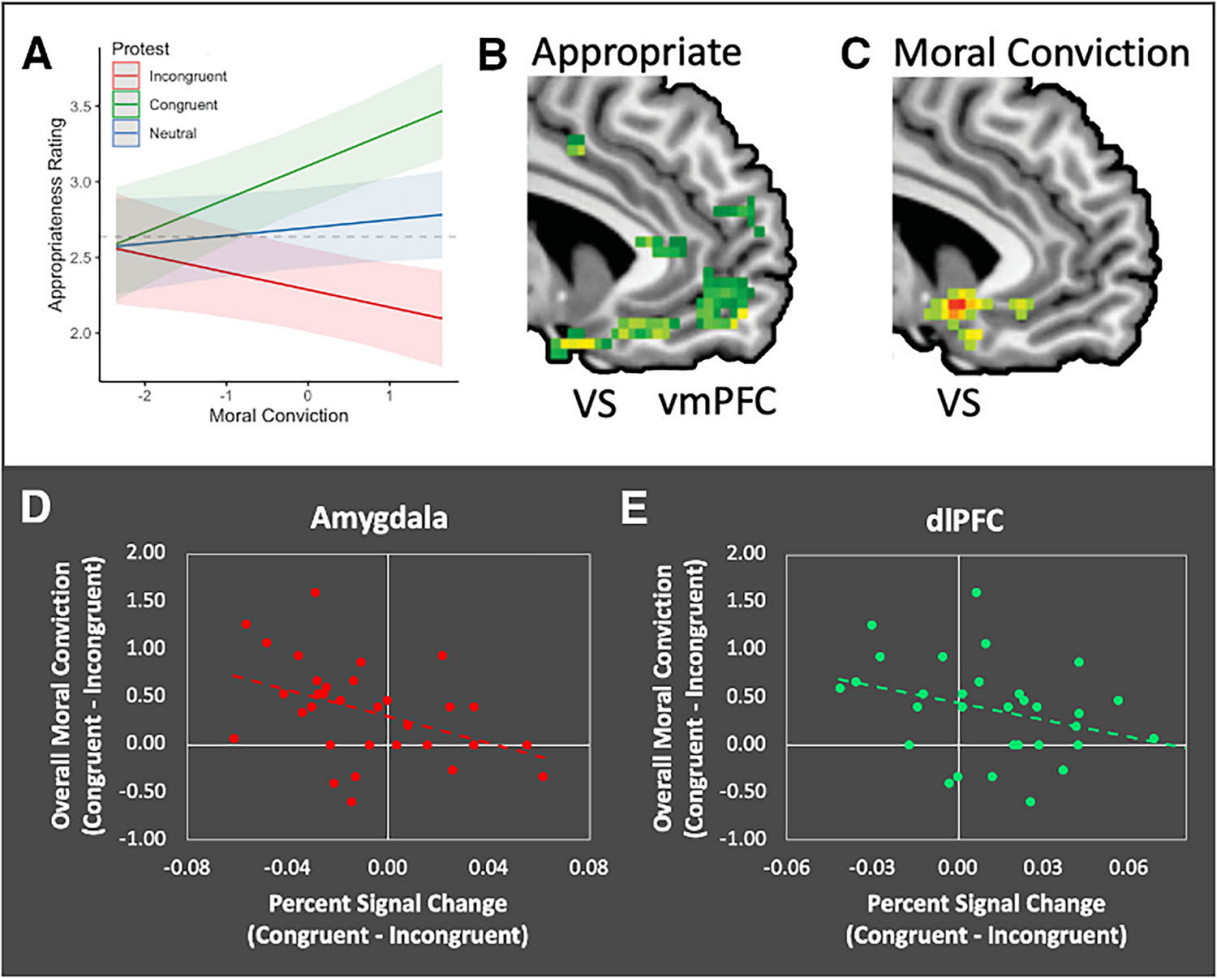
Neural response to the congruency of violent political protests. In this study, college students reported their moral convictions about a variety of sociopolitical issues (e.g., Black Lives Matter, abortion rights, ecological transition) prior to undergoing functional MRI scanning. During scanning, they were asked to evaluate the appropriateness of violent protests that were ostensibly congruent or incongruent with their views about sociopolitical issues. (A) An interaction between congruency and moral conviction predicted appropriateness ratings. Parametric increases in neurohemodynamic signal in the ventromedial prefrontal cortex (vmPFC) and ventral striatum (VS) predicted (B) appropriateness ratings and (C) the intensity of participants’ moral convictions, for congruent causes. When participants showed greater overall moral conviction (the difference in mean moral conviction for congruent vs. incongruent issues), decrease in response was detected for congruent issues in (D) amygdala and (E) dorsolateral prefrontal cortex (dlPFC). Adapted from Ref. [113].

Other studies have highlighted the role of the reward system in motivating hypothetical actions in response to moral convictions. In one study, college students were surveyed about their political affiliation and moral convictions on various sociopolitical issues (e.g., Black Lives Matter, abortion rights, or foreign aid). Later, while in the MRI scanner, these participants were asked to rate the appropriateness of photos depicting violent protests in the United States that either aligned with (congruent) or conflicted with (incongruent) their moral beliefs [113]. The effect of moral conviction on violence appropriateness was moderated by participants’ views on the issues that motivated the protests (Figure 1A). Judgments about the appropriateness of violence (Figure 1B) and judgments of moral conviction (Figure 1C) were associated with a parametric increase in neurohemodynamic activity in the vmPFC and ventral striatum when perceiving violent protests that were congruent with participants’ moral views. Overall moral conviction, operationalized as the difference between the average moral conviction ratings on congruent versus incongruent political protests, was associated with decreased activity in the amygdala, which encodes emotionally salient stimuli, and in the dlPFC, involved deliberative reasoning, when perceiving congruent versus incongruent violent protests (Figure 1D,E). Thus, perceiving congruent (“my‐side”) protests evoked increased signal in the reward circuit and decreased signal in regions involved in emotional salience and emotion regulation.

In another study, Pakistani Muslims supporting the cause of Kashmir were selected to participate based on their beliefs and support for the jihadist group fighting for the integration of this region in Pakistan [114]. They were asked to communicate in an fMRI study their willingness to fight and die for a series of values related to Islam (e.g., Sharia should be applied in all Muslim countries) and current international politics on a Likert scale of 7‐points. As in the study by Workman and colleagues [113], willingness to fight for Islamist values was associated with a parametric increase in activity in the vmPFC and a decrease in activity in the dlPFC, as well as weaker functional connectivity between the vmPFC and the dlPFC. These results suggest that motivation to fight and die for Islamist values engages brain regions associated with subjective value coding (vmPFC) rather than material cost integration (dlPFC) during decision‐making, supporting the idea that a cost−benefit calculation does not mediate decisions about costly sacrifices motivated by moral values [115]. Another study combined fMRI with the use of multidimensional scaling to investigate the perception and evaluation of political candidates when these participants were asked to read and evaluate 80 political statements [116]. A component of participants’ responses related to political radicalism was associated with a parametric increase in activity within the ventral striatum. These regions modulate the online expression of proviolence attitudes, consistent with the other work linking this brain circuit to moral beliefs and political radicalism.

To investigate the influence of the strength of political attitudes and their moral significance on the neural mechanism of decision‐making, participants in an fMRI study were scanned while deciding which of two groups of protesters, representing various sociopolitical issues, they supported more [108]. Moral conviction and support levels for each issue were assessed weeks before scanning. Support for the protesters was positively associated with hemodynamic response in the valuation network, particularly in vmPFC and amygdala. Decisions tied to stronger moral conviction elicited greater neurohemodynamic activity in the aINS, ACC, and lPFC. These findings suggest that decisions involving moral conviction increased emotional salience (anterior insula and ACC) while also engaging goal‐directed cognitive processes (lPFC). This latter region plays a role in signaling the importance of upholding moral convictions and guiding behaviors toward support for those goals [117]. Moreover, functional connectivity between lPFC and vmPFC was also stronger when moral conviction levels were higher, indicating that the lPFC operates in concert with the vmPFC in this context, consistent with the vmPFC well‐established role in weighing decision options [118].

People's reluctance to adjust morally convicted beliefs in response to factual evidence has some overlap with deontological moral reasoning. A dual‐process theory of moral judgment has been proposed, in which the vmPFC plays a key role in deontological reasoning, while processing in frontoparietal regions, such as the dlPFC, underlies utilitarian moral reasoning [119, 120]. At the same time, it has been questioned whether the underlying evidence clearly supports this theory [121, 122]. Additionally, subsequent work has emphasized the role of vmPFC in integrating emotional responses with utilitarian considerations [123, 124, 125], rather than a purely affective role.

As noted above, regarding moral conviction, the vmPFC appears to play a central role in the willingness to take aggressive action to uphold moral convictions, while dlPFC is also engaged by consideration of topics with high moral conviction. Thus, unlike in the dual process view of deontological moral reasoning, both cognitive and emotional processing seem to be activated by morally convicted beliefs [66]. Functional neuroimaging studies indeed suggest that moral convictions related to sociopolitical or economic issues are associated with a distinctive neural signature within the brain's valuation system and its functional interaction with the neural circuit supporting social cognition.

## 7 Metacognition and Moral Beliefs

Moral convictions can easily become inflexible when people believe that they represent universal moral imperatives [64, 93]. More broadly, moral convictions can reduce sensitivity to factual evidence, which can lead to rejecting arguments grounded in cost/benefit analysis. For instance, anti‐GMO (genetically modified organism) attitudes are often not grounded in an objective scientific understanding of biology. One study found that people who oppose GMOs are often moral absolutists, driven by emotional feelings of disgust, and are resistant to risk−benefit calculations or arguments based on factual data [126]. However, a subsequent series of studies suggest that most presumed absolutists do not understand the key question and/or cannot provide a valid answer to it [127]. Similarly, a study conducted in the United States, France, and Germany, using representative samples, found that individuals with the most extreme anti‐GMO attitudes were the least scientifically knowledgeable [128]. The latter findings suggest that opposition to GMOs and other forms of new technology is rooted in fear and a lack of education rather than disgust.

An emerging body of research suggests that cognitive processes play a critical role in shaping moral conviction by influencing how individuals form beliefs, evaluate evidence, and make decisions. Across ideological and political spectrums, individuals who hold extreme social, political, or religious convictions, including those who engage in ideologically motivated violence, tend to exhibit a consistent psychological profile characterized by slower uptake of new information [129]. Interestingly, ideologies and moral convictions share key features, such as absolute descriptions and prescriptions for how individuals ought to think, behave, and interact with others. They delineate what is permissible and what is forbidden, and they often prescribe punishments and social exclusion for those who deviate from established norms [130]. According to developmental research, individual differences in proneness to fascism manifest as early as in people's teenage years. Prejudiced children tend to adopt a rigid mindset that the world is split into binaries, and this rigidity is not constrained to one domain [131].

The lack of mental flexibility associated with dogmatism may reflect poor metacognition, which refers to the ability to reflect upon and evaluate our own beliefs. Metacognition encompasses self‐reflection, knowledge assessment, and self‐evaluation of learning. A central aspect of metacognition is confidence, which reflects the degree of certainty we assign to other cognitive processes. For instance, we have high confidence when we believe our thinking is valid, and low confidence when we doubt the accuracy of our knowledge. Overconfidence is more prevalent among political extremists than among political moderates. A mechanism for this relationship follows from political extremists exhibiting cognitive simplicity (perceiving the social world in black‐and‐white terms) and having less tolerance for those holding opposing beliefs [132]. Their heightened judgmental certainty about their own knowledge, regardless of actual knowledge, seems to be mediated by their belief in simple solutions to complex sociopolitical issues, such as the 2016 European Union refugee crisis [133]. This relationship between overconfidence and cognitive rigidity emphasizes the importance of metacognitive awareness in understanding how individuals process information and adjust their beliefs.

Metacognitive awareness also seems to play a role in individuals’ receptiveness or resistance to updating incomplete knowledge when faced with additional evidence [134]. A standard approach to measuring metacognition involves asking which of two perceptual fields contains more dots and then rating confidence in each judgment. The key measure is not performance on the task. In fact, an advantage of this approach is that task performance is largely deterministic, where difficulty can be easily quantified, and thus task performance can be controlled. The primary measures of interest are instead metacognitive sensitivity (meta‐d’) and metacognitive efficiency (meta‐e), which both reflect the degree to which an individual's confidence judgments track their actual performance. The key difference is that meta‐d’ is confounded by task performance, making comparisons valid only at the same performance level. In contrast, meta‐e adds a correction for task performance, thus making comparisons meaningful even when task performance differs. A high score on these measures indicates that when people believe they responded correctly, they are actually more likely to have answered correctly. Metacognitive efficiency correlates across different cognitive task domains even when task performance does not [135], supporting the idea that metacognition is a distinct cognitive process from the tasks in which it is typically measured.

**FIGURE 2:**
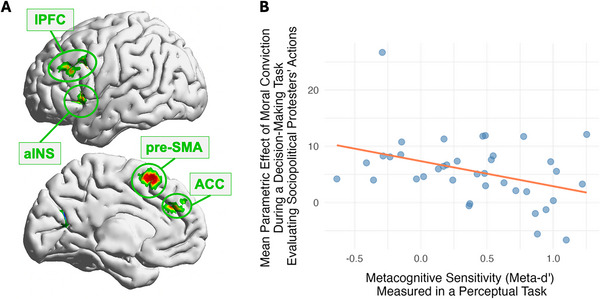
Neural activity associated with moral conviction level about sociopolitical issues (e.g., police brutality, climate change legislation, gun regulation) during decision‐making, and relationship with metacognitive sensitivity. (A) The pre‐SMA, lPFC, ACC, and aINS showed increased hemodynamic response when participants made decisions about sociopolitical issues with higher moral conviction. During fMRI scanning, participants chose which of two groups of protesters advocating for different issues they supported more. The higher of the two moral conviction ratings assigned to the issues in a given decision was used as a proxy for the moral conviction level of that decision. The figure displays the lateral and medial views of the left hemisphere. (B) Individual metacognitive sensitivity was negatively correlated with brain activity associated with moral conviction. Adapted from Ref. [108].

Several studies have linked metacognitive abilities with how people approach sociopolitical topics. Individuals with poor metacognitive performance tend to be more dogmatic in their self‐reported sociopolitical beliefs [136]. Such individuals are particularly unlikely to be influenced by population attitudes when they have a morally convicted belief [71]. Furthermore, in a functional neuroimaging study examining decisions about sociopolitical issues, moral conviction elicited a stronger brain response in participants with poorer metacognitive ability (Figure 2) [108]. While moral conviction was associated with greater brain response (in presupplementary motor area, lPFC, ACC, and anterior insula), this effect was particularly strong in those with poor perceptual metacognition. Relatedly, individuals who exhibited heightened neural response to moralized information also made faster decisions when they held stronger moral convictions about the protest topics, suggesting an interconnection between metacognition, moral conviction, and decision‐making. Importantly, in both this study and an earlier one [71], metacognitive sensitivity was measured using a low‐level perceptual task. The finding that such a low‐level cognitive process seemingly unrelated to moral or political reasoning predicts both behavioral and neural responses to sociopolitical issues suggests a fundamental role of metacognition in shaping belief rigidity and moral conviction. Further illustrating the interplay between sociopolitical views and metacognition, a recent reanalysis of data from a 6‐month longitudinal study involving 1191 participants found that conservatives, in particular, demonstrated lower metacognitive efficiency when evaluating the accuracy of news stories that conflicted with their ideological beliefs [137]. These results complement the research on individual differences in metacognition, suggesting that metacognitive efficiency is shaped not only by stable traits, with downstream effects on sociopolitical decisions, but also by contextual factors, such as ideological congruency.

Individual differences in metacognition appear to be associated with the tendency to hold rigid moral beliefs. Intriguingly, new research on twins found that shared environmental factors predict higher‐order cognitive abilities such as metacognitive sensitivity and mentalizing, while genetic factors are more prominent in lower‐order measures, including both task performance and mean level of confidence [138]. Another study examining specific polymorphisms in genes related to dopamine and serotonin function found a clear effect of a serotonin transporter gene on bias in level of confidence, but no conclusive evidence for the impact of genotype on metacognitive efficiency [139]. The lack of evidence for heritability suggests that metacognitive sensitivity can be improved through factors such as learning, personal experience, and external guidance.

It is also possible that moral decisions are associated with impaired metacognition when individuals hold them with strong conviction. There is evidence in the domain of climate change that metacognitive efficiency for responses to factual questions is reduced, compared to an individual's baseline performance, when the topic is morally charged [140]. Extreme views and beliefs are often held with strong confidence, which can predispose individuals to moralize and contribute to polarization. A series of studies reported that confidence in a first‐order belief correlates with the extremity of that belief across various participant samples and tasks [141]. These findings can be considered in conjunction with prior work that demonstrated that people tend to treat high confidence as evidence of accuracy [142]. Together, they provide a possible mechanism by which extreme views are more persuasive and spread further due to being expressed confidently, contributing to increased dogmatism and polarization in society.

In sum, the ability to reflect on, evaluate, and control cognitive processes plays a crucial role in shaping moral convictions. Poor metacognitive abilities appear to be linked to a reduced capacity to recalibrate beliefs and attitudes when they are moralized [71, 93], thereby reinforcing confirmation and myside biases. However, the specific interplay between moral conviction and metacognition remains unclear. It is possible that strong moral convictions on sociopolitical issues hinder reflective thinking across domains. Conversely, low trait‐level metacognitive efficiency may predispose individuals to adopt rigid moral beliefs, with moral conviction representing just one manifestation of a broader cognitive pattern. Another possibility is that both phenomena reinforce each other. Finally, metacognitive abilities and rigid moral convictions may both be influenced by an underlying third variable, yet to be identified. Regardless of the specific mechanisms, given the apparent association between rigid moral beliefs and poor metacognition, and the malleability of metacognition, future studies should examine whether interventions aimed at enhancing metacognition could mitigate the potentially detrimental effects of strong moral convictions. Considering extensive research on improving metacognition in educational settings [143, 144], articulating the relationship between metacognitive failure, moralization, and dogmatism offers a promising pathway for developing interventions aimed at mitigating adverse outcomes.

## 8 Information Seeking and Avoidance

Moralization often intensifies beliefs to the point that they become dogmatic, a phenomenon associated with poor metacognitive abilities. Avoidance of relevant information may represent a key intermediate link between poor metacognition and maintenance of dogmatic beliefs. It has long been recognized that individuals may avoid information if paying attention to it would induce mental discomfort or cognitive dissonance. As discussed further below, individuals with poor metacognitive abilities are more likely to avoid new information that challenges existing beliefs and decisions [145].

One early description of information avoidance came from Maslow [146], who suggested that “we can seek knowledge to reduce anxiety, and we can also avoid knowing in order to reduce anxiety.” Similar ideas also emerged from research on cognitive dissonance [147], also known as *selective exposure*. Specifically, there is strong evidence that individuals exhibit a “defensive motivation” to pay more attention to information that is congenial with their pre‐existing beliefs than to information that challenges those views. At the same time, there is also a countervailing and often weaker motivation to be accurate in their beliefs, which also plays a role in information search [148]. More recent work has shown that individual differences in self‐reported receptiveness to opposing views are associated with various real and hypothetical measures of interest in and engagement with opposing political viewpoints, and with how negatively people evaluate others with opposing viewpoints [149]. In a similar vein, political ideology, which comprises a set of truths and moral convictions that people live by and share with others [130], can bias and constrain individuals’ cognitive thinking. Specifically, one study found that political ideology impairs logical reasoning for political content (such as gun control, capital punishment, and immigration) but not for politically irrelevant syllogisms (concerning animals, plants, and objects) [150]. An example of the type of logical syllogism being judged in these studies is as follows: “All drugs that are dangerous should be illegal. Marijuana is a drug that is dangerous. Therefore, marijuana should be illegal.” Consistent with prior work on belief bias and motivated reasoning, individuals on the ideological left are more accurate in judging valid liberal conclusions and rejecting invalid conservative conclusions, whereas the reverse is true for conservatives. Subsequent work replicated this finding across both American and German samples. Additionally, it was robust even with monetary incentives for accuracy, which should motivate people to judge the logic as accurately as they can, regardless of whether they agree with its conclusion [151]. These findings suggest that a preference for engaging with congenial information and a bias in favor of believing such information could contribute to real‐world political polarization.

Information avoidance is a related but distinct concept from selective exposure, whereby people actively avoid learning new information that is likely to be aversive or unpleasant. Selective exposure occurs after the content of the information is known, while information avoidance is a decision made prior to exposure [152]. One recent theory suggests that the norepinephrine system plays a critical role in information avoidance [153]. This work elaborates on earlier models describing distinct types of utility, including instrumental utility (extrinsic rewards) and hedonic utility (positive affect), that can motivate information seeking [154]. Tendencies to seek versus avoid information are predicted by different trait factors, suggesting that they are not simply the inverse of one another despite surface‐level appearances that they would be [155]. Information‐seeking in safe environments is driven by the dopaminergic system. However, contextual and trait factors, such as anxiety, intolerance for uncertainty, or a need for cognitive closure, can make people feel overwhelmed by, rather than curious about, new information. This negative signal, driven by noradrenergic activity originating in locus coeruleus and amygdala, is theorized to counteract the competing drive to seek out new information.

One prior study examined the brain response when processing challenges to strongly held political and nonpolitical beliefs [156]. Challenges to political beliefs more strongly activate regions of the default‐mode network (anterior medial prefrontal cortex, posterior cingulate cortex, and angular gyrus), highlighting the self‐relevance of these topics. Individuals who were more resistant to changing beliefs showed greater amygdala activity while processing those challenges. Finally, within individuals, resistance to belief change was associated with more activity in the dorsomedial PFC and less activity in the orbitofrontal cortex. These findings are consistent with the idea that information avoidance, mediated by neural activity in the amygdala and other noradrenergic regions [153], may be a key mechanism by which people resist challenges to morally convicted beliefs.

Finally, there is evidence that people rely on confidence to guide information‐seeking and information avoidance decisions. In other words, when subjective confidence is low, people are more likely to seek out new information likely to improve subsequent performance. This relationship was initially observed in the domain of perceptual decisions, where confidence is typically well‐aligned with accuracy [157]. This effect was also stronger in individuals with higher metacognitive efficiency, suggesting that those who more accurately assess confidence rely on it the most in decision‐making. However, another recent study shows that the relationship holds even for more complex judgments in which confidence is very weakly related to accuracy. Specifically, French participants were asked to evaluate whether news headlines on contentious topics not directly about political parties (democracy, social justice, and ecology) were true or false [158]. Judgment accuracy varied greatly between different topics, yet confidence was only weakly related to accuracy. Still, when participants were given the opportunity to pay a small fee to access or avoid new information that confirmed or debunked the headline, they were significantly more likely to want this information when they were less confident, especially for items that they judged to be false.

Interestingly, the relationship between confidence and information‐seeking tends to be weaker in those with a tendency toward dogmatic beliefs. Specifically, individuals who score high in dogmatism are more reluctant to pay to access information that will lead to a more accurate decision on a perceptual task, particularly when confidence is low [159]. Other studies have shown that more dogmatic individuals, as well as those with higher confidence and lower domain‐general metacognitive sensitivity, are less likely to update inaccurate beliefs about climate change when presented with new information that challenges climate change skepticism [145]. Furthermore, the relationship between metacognition (overconfidence and low metacognitive insight) with belief updating was mediated by the degree of climate change skepticism, with inaccurate metacognition predicting higher skepticism, and more skepticism predicting lower levels of belief updating. Still, it is notable that dogmatism and poor metacognition predict resistance to corrective new information not only in a controversial political domain but also when decisions involve meaningless perceptual stimuli. Thus, this relationship has strong generality.

Thus, an important research direction involves investigating the role of information avoidance in moral conviction. Specifically, do individuals tend to avoid information more when confronted with information that challenges their moral convictions? These processes also suggest the involvement of specific brain mechanisms, with amygdala and/or other salience mechanisms theorized to underlie information avoidance. It is essential to also examine how the engagement of these mechanisms influences the likelihood of updating moralized beliefs when presented with countervailing evidence.

## 9 Conclusion

Morality evolved to facilitate cooperation and group living, playing an integral role in cultural evolution. Moral convictions have undoubtedly contributed to many important social and political advancements throughout history by motivating greater civic engagement. However, it is important to recognize that morality is also deeply rooted in coalitional dynamics and group identity. As such, especially when moral values are held with strong conviction, morality can exacerbate social tensions and have many maladaptive consequences, such as dogmatism, intolerance, and symbolic violence or even physical violence, which at times outweigh its benefits. When an issue becomes moralized, it tends to attract greater attention from individuals and institutions. Yet, this process also risks inflaming people's righteous indignation, making rational deliberation and compromise more difficult and often fueling affective polarization.

Interdisciplinary research presented in this article is crucial for understanding the functional architecture of moral conviction and can help clarify when moralization drives societal progress and when it hinders it. Given the potential for moral conviction to obstruct constructive solutions, we should be cautious about moralizing political, public health, and economic issues—such as climate change, refugees, prison reform, vaccination, and drug addiction—to remain open to pragmatic considerations and engage in rational cost−benefit analysis.

The body of work reviewed here has begun to elucidate the proximate mechanisms underlying moral conviction. Perceptions of moral violations activate both the emotional saliency system and the valuation system. Moreover, poor metacognitive abilities have been associated with a stronger influence of moral conviction on both behavioral and brain responses. Understanding how moralization interacts with other aspects of cognition is valuable to social scientists and policymakers seeking to promote more constructive forms of collective action. Moral language, with its strong connections to emotion and value, is among the most effective forms of persuasion. However, its persuasive force can have unintended consequences, including political polarization and escalation of conflict in international relations, and in some cases, warfare [160]. Thus, when encountering highly moralized attitudes and beliefs, it may be helpful to consider whether they stem from differing moral motivations, the social relationships or group status they aim to regulate, or the coalitional interests they serve.

Importantly, we are not asserting that moral convictions are inherently harmful, that compromise is always preferable, or that all forms of violence are unjustified. Instead, in politics, the argument for compromise rests on its instrumental value [161]. Compromises can lead to both desirable and undesirable outcomes. Yet, a political system that consistently resists compromise, where leaders insist only one outcome is morally legitimate and treat disagreement as a moral failure, runs the risk of fueling bitterness and resentment. In a pluralistic society, wisdom often requires prioritizing values like tolerance, mutual respect, and political stability over narrower ideological commitments.

## Author Contributions

J.D. conceived and wrote the article, M.S.C. wrote the article, Q.C. wrote the article.

## Conflicts of Interest

The authors declare no competing interests.
